# Combined effects of ARNI and SGLT2 inhibitors in diabetic patients with heart failure with reduced ejection fraction

**DOI:** 10.1038/s41598-021-01759-5

**Published:** 2021-11-16

**Authors:** Hyue Mee Kim, In-Chang Hwang, Wonsuk Choi, Yeonyee E. Yoon, Goo-Yeong Cho

**Affiliations:** 1 Division of Cardiology, Department of Internal Medicine, Chung-Ang University Hospital, Chung-Ang University College of Medicine, Seoul, South Korea; 2 Department of Cardiology, Cardiovascular Center, Seoul National University Bundang Hospital, Seongnam, Gyeonggi South Korea; 3 Department of Internal Medicine, Seoul National University College of Medicine, Seoul, South Korea; 4 Cardiovascular Center, Sheikh Khalifa Specialty Hospital, Ras Al Khaimah, United Arab Emirates

**Keywords:** Cardiology, Heart failure

## Abstract

Angiotensin receptor-neprilysin inhibitor (ARNI) and sodium–glucose co-transporter-2 inhibitor (SGLT2i) have shown benefits in diabetic patients with heart failure with reduced ejection fraction (HFrEF). However, their combined effect has not been revealed. We retrospectively identified diabetic patients with HFrEF who were prescribed an ARNI and/or SGLT2i. The patients were divided into groups treated with both ARNI and SGLT2i (group 1), ARNI but not SGLT2i (group 2), SGLT2i but not ARNI (group 3), and neither ARNI nor SGLT2i (group 4). After propensity score-matching, the occurrence of hospitalization for heart failure (HHF), cardiovascular mortality, and changes in echocardiographic parameters were analyzed. Of the 206 matched patients, 92 (44.7%) had to undergo HHF and 43 (20.9%) died of cardiovascular causes during a median 27.6 months of follow-up. Patients in group 1 exhibited a lower risk of HHF and cardiovascular mortality compared to those in the other groups. Improvements in the left ventricular ejection fraction and E/e′ were more pronounced in group 1 than in groups 2, 3 and 4. These echocardiographic improvements were more prominent after the initiation of ARNI, compare to the initiation of SGLT2i. In diabetic patients with HFrEF, combination of ARNI and SGT2i showed significant improvement in cardiac function and prognosis. ARNI-SGLT2i combination therapy may improve the clinical course of HFrEF in diabetic patients.

## Introduction

In recent years, innovative developments have been made in the management of heart failure (HF), based on robust evidence from landmark trials of angiotensin receptor-neprilysin inhibitors (ARNI) and sodium–glucose co-transporter-2 inhibitors (SGLT2i)^1–3^. In addition to acting as blockers of the renin-angiotensin system (RAS), ARNI also inhibit neprilysin, which enhances the function of the natriuretic peptide system, causing vasodilation, natriuresis, inhibition of myocardial remodeling and sympathetic nerve suppression^4^. ARNI reduces cardiovascular mortality and hospital admissions in patients with heart failure with reduced ejection fraction (HFrEF), regardless of the presence of diabetes. They have been shown to results in left ventricular (LV) reverse remodeling, with decreased levels of N-terminal probrain natriuretic peptide (NT-proBNP)^5,6^. SGLT2i, which were primarily developed as anti-diabetic drugs, have been demonstrated to decrease the risk of cardiovascular events in large-scale clinical trials. The cardiovascular benefits of SGLT2i is mainly due to the reduction of hospitalization for HF (HHF), which is suggested to be derived from its natriuresis and osmotic diuresis effects, together with improvements in cardiac metabolism and bioenergetics^7–9^.

Since ARNI and SGLT2i have different mechanisms of action in the treatment of HF, the concurrent use of drugs from these two classes may have additive or synergistic effects in improving myocardial function and cardiovascular prognosis. Sub-analyses of recent trials have suggested that the benefits of ARNI and SGLT2i are independent of each other^3,10,11^. However, there are limited studies investigating the prognosis and the changes in cardiac function in patients treated with a combination of ARNI and SGLT2i. Therefore, this study aimed to investigate whether a combination of an ARNI and a SGLT2i could be more effective in improving cardiac function and disease prognosis in diabetic patients with HFrEF.

## Methods

### Study population

**Figure 1:**
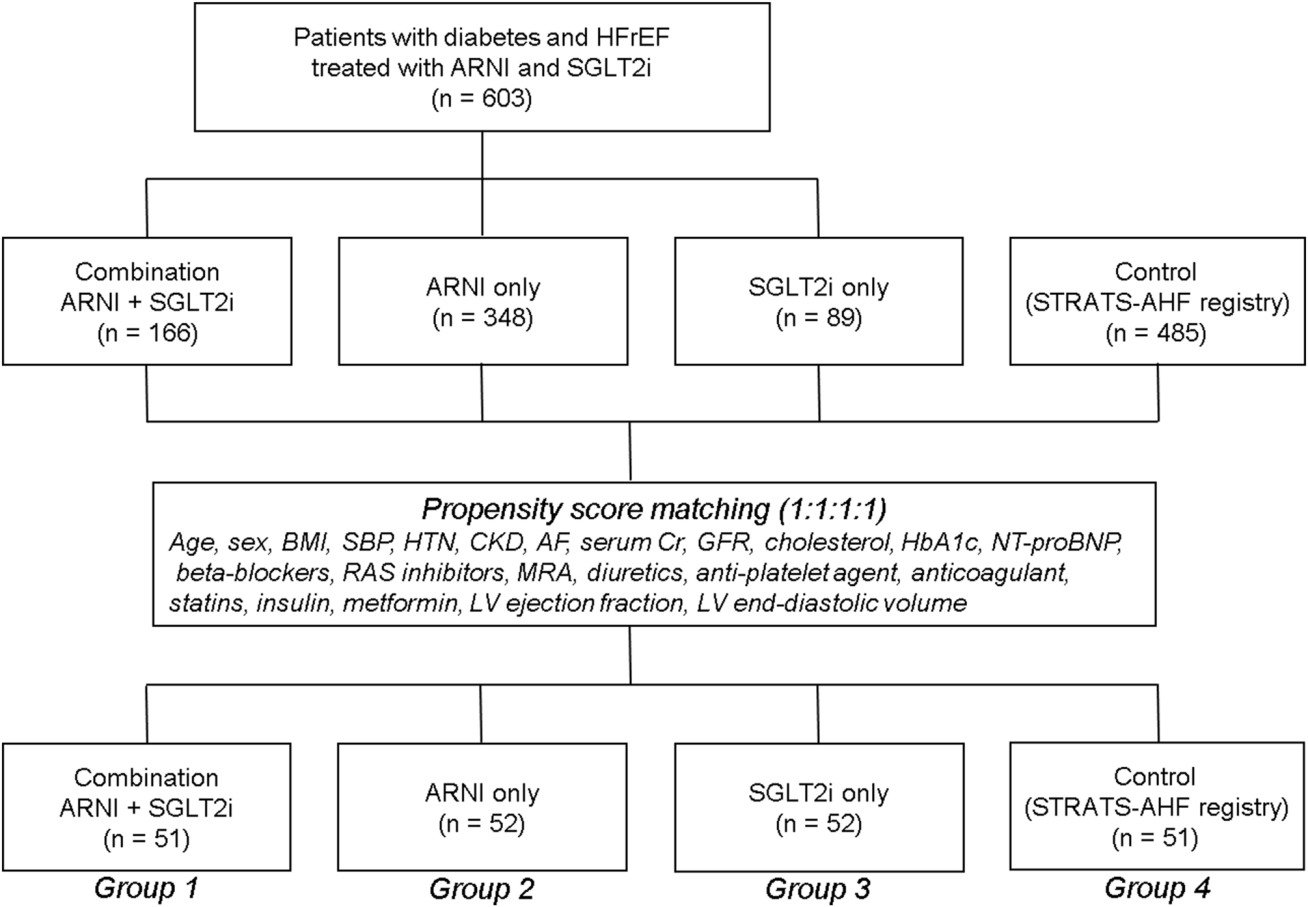
Flowchart of the study population.

The study included patients registered as inpatients or outpatients for the treatment of HF at Seoul National University Bundang Hospital and Chung-Ang University Hospital. Diabetic patients with an LV ejection fraction (LV-EF) < 40%, who had been prescribed an ARNI and/or a SGLT2i at either of the two hospitals between October 2017 and December 2020 were included. Diabetic patients with an LV-EF < 40% from The Strain for Risk Assessment and Therapeutic Strategies in patients with Acute Heart Failure (STRATS-AHF) registry (N = 4312), who were prescribed RAS blockers, but not ARNI and SGLT2i, were included as controls for the study. Details of the STRATS-AHF registry have previously been described^12^. The patients were divided into 4 groups based on the treatment prescribed: group 1 (combination of ARNI and SGLT2i, N = 166), group 2 (ARNI only, N = 348), group 3 (SGLT2i only, N = 89), group 4 (neither, N = 485) (Supplementary Table 1). Propensity score matching in a 1:1:1:1 ratio for age, sex, body mass index, systolic blood pressure, hypertension, chronic kidney disease, atrial fibrillation, creatinine, glomerular infiltration rate, total cholesterol, hemoglobin A1c protein, NT-proBNP, LV-EF and LV end-diastolic volume (LV-EDV), and the use of beta blockers, RAS blockers, mineralocorticoid receptor antagonists (MRA), loop diuretics, antiplatelet drugs, oral anticoagulants, statins, insulin, and metformin was used to select a total of 206 patients for the study (group 1, N = 51; group 2, N = 52; group 3, N = 52; group 4, N = 51). This data is summarized in Fig. 1.

The study protocol was approved and the written informed consent was waived by the Seoul National University Bundang Hospital Institutional Review Board (IRB No. B-2101-661-107) and the Chung-Ang University Hospital Institutional Review Board (IRB No. 2101-003-19348), given the retrospective nature of this study. All clinical investigations were conducted according to the principles of the Declaration of Helsinki.

### Echocardiography

Echocardiographic assessments were conducted in accordance with the American Society of Echocardiography guideline^13^. LV-EDV, LV end systolic volume (LV-ESV), and LV-EF were calculated as per the biplane Simpson method. LV mass index (LVMI) was calculated using Devereux’s formula^14^. Peak early (E) and late (A) diastolic mitral inflow velocities and deceleration time were also measured. Peak systolic (s′), early (e′), and late diastolic (a′) velocities at the septal mitral annulus were recorded by Doppler imaging. Left atrial (LA) volumes were determined using the biplane area-length method and used to calculate the LA volume indexes (LAVI). Right ventricular systolic pressure was estimated based on the peak velocity of tricuspid regurgitation.

### Outcomes

Our study patients were followed up until March 2021. HHF and cardiovascular death were recorded for the assessment of outcomes. HHF was defined as hospitalization for worsening signs or symptoms of HF, requiring the administration of intravenous diuretics or vasodilators. The data on mortality and cause of death were obtained from the hospital records, the Korean Ministry of Security and Public Administration, and the National Statistical Office of Korea. The index date of each group was defined as follow: group 1, the date of prescription of both ARNI and SGLT2i; group 2, the date of first prescription of ARNI; group 3, the date of first prescription of SGLT2i; and group 4, the date of hospitalization for acute HF. Echocardiographic data, including LV-EF, LV-EDV. LV-ESV, LV-end diastolic dimension (LV-EDD), LV-end systolic dimension, LVMI, LAVI and mitral E/e′ were evaluated at the baseline, and after 1–6 months, 6–12 months, and 12–24 months. The data for the four groups was compared. For patients in group 1, serial echocardiograms were assessed based on the order in which treatment with the ARNI or SGLT2i was initiated.

### Statistical analysis

The baseline characteristics of the patients are described as numbers and percentages for categorical variables, and as mean ± standard deviation or median with interquartile range for continuous variables. Comparisons between the groups were conducted using one-way analysis of variance (ANOVA) or Kruskal Wallis test. Pearson’s Chi-squared test was used for the comparison of categorical variables. Changes in the echocardiographic parameters were compared using the linear mixed models, and all baseline characteristics were used as covariates. Event-free survival analyses were conducted by Kaplan–Meier method with log-rank test and multivariable Cox proportional hazard model adjusted with baseline characteristics including NT-ProBNP. All statistical analyses were performed using SPSS 22.0 (SPSS Inc., Chicago, IL, USA) and R programming software version 3.6.1 (The R Foundation for Statistical Computing, Vienna, Austria). Differences were considered statistically significant when P < 0.05.

## Results

### Baseline characteristics and echocardiographic measurements

Baseline characteristics of the propensity score-matched study population are summarized in Table 1. The mean age of the study population was 67.0 ± 12.0 years and 68% of the patients were male. There were no significant differences in the prevalence of underlying diseases between the subgroups. All patients were receiving treatment with RAS blockers (including ARNI), 86.4% were receiving beta blockers, and 45.6% were receiving MRA. For glycemic control, 70.4% of the patients were being treated with metformin, 33% with insulin, and 35.4% with sulfonylureas. Laboratory findings, including hemoglobin A1c, creatinine, and estimated glomerular filtration rate were not significantly different between the four groups. However, differences were noted in the NT-proBNP and hemoglobin.

**Table 1:** Baseline characteristics according to the groups.

	ARNI + SGLT2i (Group 1, N = 51)	ARNI only (Group 2, N = 52)	SGLT2i only (Group 3, N = 52)	Control (Group 4, N = 51)	P value
**Demographics**					
Age (years)	67.1 ± 11.7	67.5 ± 12.1	67.8 ± 112.8	68.5 ± 111.4	0.452
Male (N, %)	38 (74.5%)	34 (65.4%)	37 (71.2%)	31 (60.8%)	0.455
Body mass index (kg/m^2^)	25.1 ± 13.7	25.2 ± 4.0	25.1 ± 3.7	24.9 ± 3.6	0.616
Body surface area (m^2^)	1.7 ± 10.2	1.7 ± 10.2	1.8 ± 10.2	1.7 ± 0.2	0.357
**Hemodynamics**					
Systolic blood pressure (mmHg)	120.8 ± 15.8	121.8 ± 17.9	120.4 ± 17.7	122.9 ± 21.3	0.900
Diastolic blood pressure (mmHg)	71.4 ± 12.9	71.9 ± 11.8	71.3 ± 18.9	75.1 ± 16.3	0.378
**Underlying diseases (N, %)**					
Hypertension	34 (66.7%)	33 (23.6%)	31 (22.1%)	42 (30.0%)	0.071
Dyslipidemia	40 (78.4%)	46 (88.5%)	42 (80.8%)	43 (84.3%)	0.552
Chronic kidney disease	18 (35.3%)	19 (36.5%)	17 (32.7%)	20 (39.2%)	0.920
Coronary artery disease	21 (41.2%)	26 (50.0%)	25 (48.1%)	28 (54.9%)	0.576
Atrial fibrillation	17 (33.3%)	17 (32.7%)	19 (36.5%)	14 (27.5%)	0.802
**Medication**					
ARB	51 (100.0%)	51 (100.0%)	38 (73.1%)	29 (56.9%)	–
ACE inhibitors	–	–	14 (26.9%)	22 (43.1%)	–
Beta blocker	44 (86.3%)	48 (92.3%)	45 (86.5%)	41 (80.4%)	0.374
MRA	26 (51.0%)	22 (42.3%)	23 (44.2%)	23 (45.1%)	0.833
Metformin	40 (78.4%)	32 (61.5%)	41 (78.8%)	32 (62.7%)	0.080
Insulin	13 (25.5%)	18 (34.6%)	11 (21.2%)	26 (51.0%)	0.007
Sulfonylurea	15 (29.4%)	13 (25.0%)	21 (40.4%)	24 (47.1%)	0.077
Antiplatelet	36 (70.6%)	38 (73.1%)	35 (67.3%)	46 (90.2%)	0.035
Anticoagulant	18 (35.3%)	17 (32.7%)	20 (38.5%)	20 (39.2%)	0.896
Statin	40 (78.4%)	45 (86.5%)	42 (80.8%)	43 (84.3%)	0.706
**Laboratory examination**					
Hemoglobin (g/dL)	13.0 ± 2.1	13.2 ± 2.1	13.9 ± 2.3	13.2 ± 2.2	0.029
Hemoglobin A1c (%)	7.0 ± 0.8	6.8 ± 0.9	7.1 ± 0.9	7.2 ± 1.4	0.775
Creatinine (mg/dL)	1.1 ± 0.3	1.1 ± 0.4	1.0 ± 0.4	1.1 ± 0.4	0.517
Estimated glomerular filtration rate (mL/min/1.73 m^2^)	72.2 ± 22.1	70.6 ± 23.3	75.8 ± 27.6	68.8 ± 24.3	0.838
Total Cholesterol (mg/dL)	150.9 ± 38.9	148.4 ± 47.8	147.7 ± 32.9	143.9 ± 35.7	0.273
NT-proBNP (pg/mL)	1319.0 (407.0–4606.6)	2692.0 (913.7–6124.4)	1007.3 (282.7–4036.0)	4037.0 (1684.0–10,742.4)	< 0.001

*ARNI* angiotensin receptor-neprilysin inhibitor, *SGLT2i* sodium–glucose co-transporter-2 inhibitors, *MRA* mineralocorticoid receptor antagonist, *BNP* B-type natriuretic peptide.

The baseline echocardiographic parameters of the patients are summarized in Table 2. At baseline, the study population showed a mean LV-EDD, LV-EDV, and LV-EF of 59.8 ± 7.6, 150.6 ± 55.7 mL, and 29.1 ± 8.6%, respectively. The mean LVMI, LAVI, ad E/e′ were 142.5 ± 41.5 mg/m^2^, 54.1 ± 23.6 mL/m^2^, and 21.6 ± 13.0, respectively. No significant differences were noted in the echocardiographic findings between the groups, except for the LVMI.

**Table 2:** Baseline echocardiographic parameters.

	ARNI + SGLT2i (Group 1, N = 51)	ARNI only (Group 2, N = 52)	SGLT2i only (Group 3, N = 52)	Control (Group 4, N = 51)	P value
LV-EDD (mm)	60.2 ± 6.7	60.0 ± 7.0	56.9 ± 8.4	60.7 ± 8.9	0.209
LV-ESD (mm)	50.5 ± 8.8	50.1 ± 8.2	47.3 ± 9.8	50.6 ± 10.0	0.444
LV-EDV (mL)	151.0 ± 59.9	153.5 ± 56.7	130.9 ± 48.4	158.3 ± 59.6	0.238
LV-ESV (mL)	109.4 ± 46.2	112.5 ± 51.1	91.2 ± 41.2	116.5 ± 51.9	0.189
LV-EF (%)	29.4 ± 9.5	28.3 ± 6.4	32.6 ± 10.6	27.7 ± 8.0	0.107
LV mass index (g/m^2^)	142.7 ± 34.4	137.2 ± 35.9	123.4 ± 19.1	159.5 ± 56.2	0.003
LA volume index (mL/m^2^)	60.3 ± 24.5	47.6 ± 26.7	60.3 ± 20.1	50.7 ± 18.4	0.018
Mitral annular e′ velocity (cm/s)	4.7 ± 1.7	4.5 ± 2.0	5.0 ± 2.4	4.6 ± 2.0	0.714
Mitral annular s′ velocity (cm/s)	4.7 ± 3.1	4.4 ± 1.1	4.8 ± 1.3	4.9 ± 1.9	0.734
Mitral annular E/e′ ratio	21.9 ± 14.0	22.0 ± 14.8	21.0 ± 11.4	21.6 ± 13.0	0.978
PASP (mmHg)	43.5 ± 14.4	39.3 ± 17.7	42.0 ± 14.6	37.8 ± 15.1	0.397
Global longitudinal strain (%)	7.5 ± 2.2	7.5 ± 2.4	7.6 ± 2.6	6.8 ± 2.5	0.264

*LV* left ventricle, *EDD* end diastolic dimension, *ESD* end systolic dimension, *EDV* end diastolic volume, *ESV* end systolic volume, *EF* ejection fraction, *PASP* pulmonary artery systolic pressure. The absolute value |x| of strain is used.

### Study outcomes

**Figure 2:**
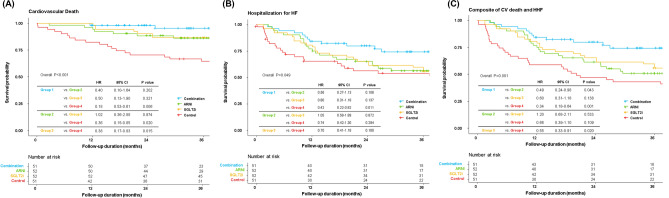
Event-free survival curves according to the groups. Kaplan–Meier curves comparing the risk of (**A**) cardiovascular (CV) death, (**B**) hospitalization for HF (HHF), and (**C**) composite of CV death and HHF, between the 4 groups.

During a median 27.6 months (interquartile range 13.6–38.0 months) of follow-up, there were 92 (44.7%) instances of HHF and 43 (20.9%) cardiovascular deaths: in groups 1, 2, 3, and 4, there were 13 (25.5%), 22 (42.3%), 26 (50.0%), and 31 (60.8%) patients who experienced HHF, and 3 (5.9%), 7 (13.5%), 9 (17.3%), and 24 (47.1%) patients who died of cardiovascular causes, respectively. The event-free survival curves are plotted for each group, and the risk of clinical outcomes was compared using the multivariable Cox proportional hazard model adjusted with baseline characteristics including NT-ProBNP (Fig. 2). Compared to the patients in group 4, those in group 1 showed a significantly lower risk of cardiovascular death (hazard ratio (HR): 0.18, 95% confidence interval (CI): 0.53–0.61, P = 0.006), HHF (HR: 0.42, 95% CI: 0.22–0.82, P = 0.011), and their composite (HR: 0.34, 95% CI: 0.18–0.64, P = 0.001). Although statistically not significant, the patients in groups 2 and 3 showed a smaller number of events than those in group 4. Overall, the probability of event-free survival decreased in the order of the group using both ARNI and SGLT2i (group 1), the groups using one of ARNI or SGLT2i (groups 2 and 3), and the control group (group 4).

### Comparison of serial echocardiographic measurements

**Figure 3:**
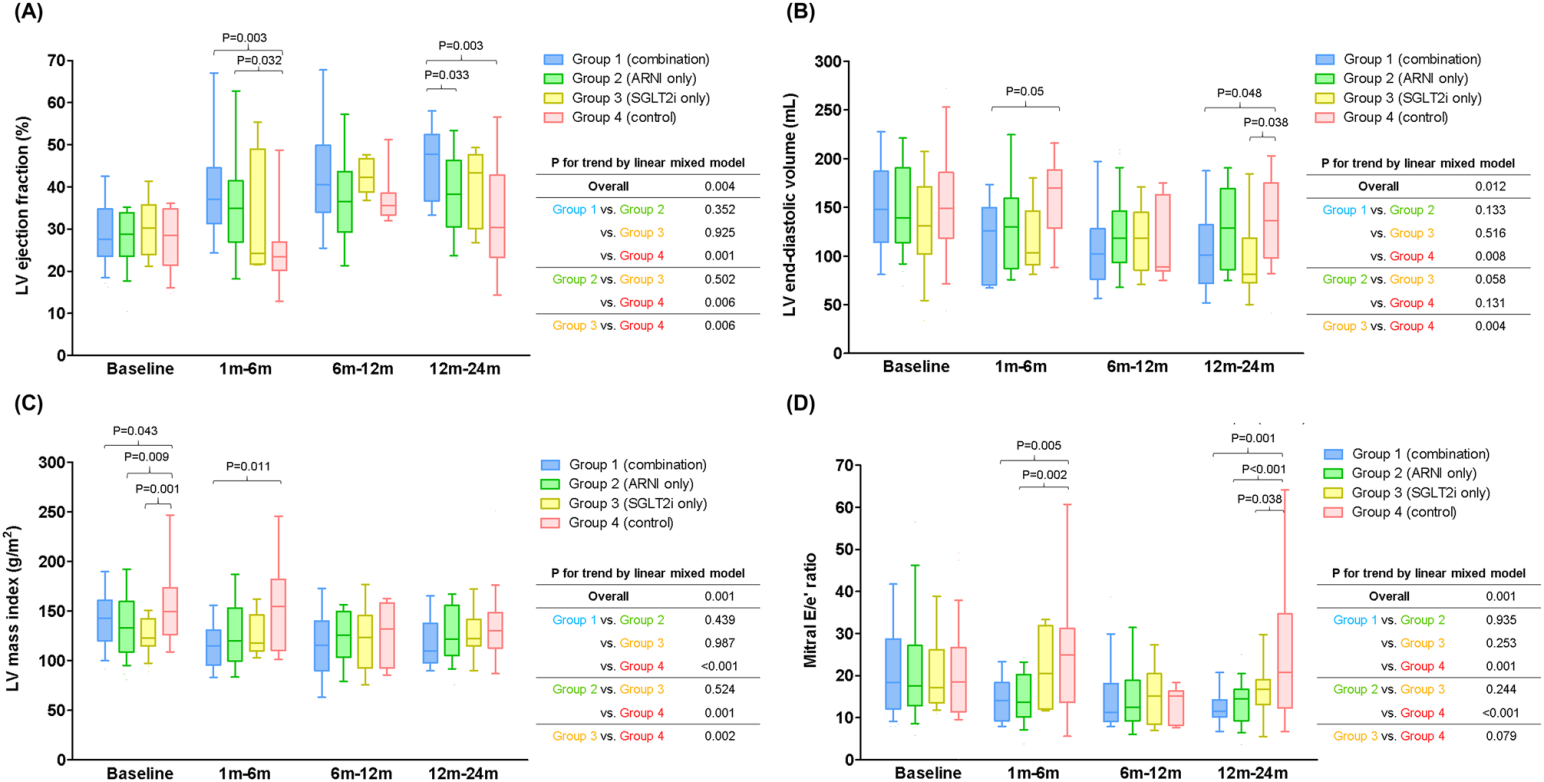
Changes of serial echocardiographic measurements based on groups. The serial measurements of (**A**) LV ejection fraction, (**B**) LV end-diastolic volume, (**C**) LV mass index, and (**D**) mitral E/e′ ratio are plotted according to the groups.

The time trajectories of LV-EF, LV-EDV, LV-MI, and mitral E/e′ ratio significantly differed between the 4 groups: the overall p-for-trends by linear mixed models were 0.004, 0.012, 0.001, and 0.001, respectively (Fig. 3). These echocardiographic parameters showed significant improvements in groups 1 to 3, compared to those in group 4. Then, we further assessed the inter-group differences at specific time intervals. The LV-EF of patients in groups 1 and 2 improved more rapidly in the 1–6 months after the initiation of treatment, compared to those in group 4 (group 1 vs. group 4, P = 0.003; group 2 vs. group 4, P = 0.032). A significant reduction in the mitral E/e′ ratio was observed in the groups 1 and 2 within 1–6 months of treatment initiation, but not in group 4 (group 1 vs. group 4, P = 0.005; group 2 vs. group 4, P = 0.002). After 12–24 months of treatment, the LV-EF and mitral E/e′ of patients in group 1 improved significantly compared to that of patients in group 4. The patients in group 1 tended to show higher LV-EF and lower mitral E/e′ ratio than those in groups 2, 3, and 4, throughout the follow-up period. However, these differences were not statistically significant.

### Changes in cardiac function based on the order initiation of ARNI and SGLT2i treatment

**Figure 4:**
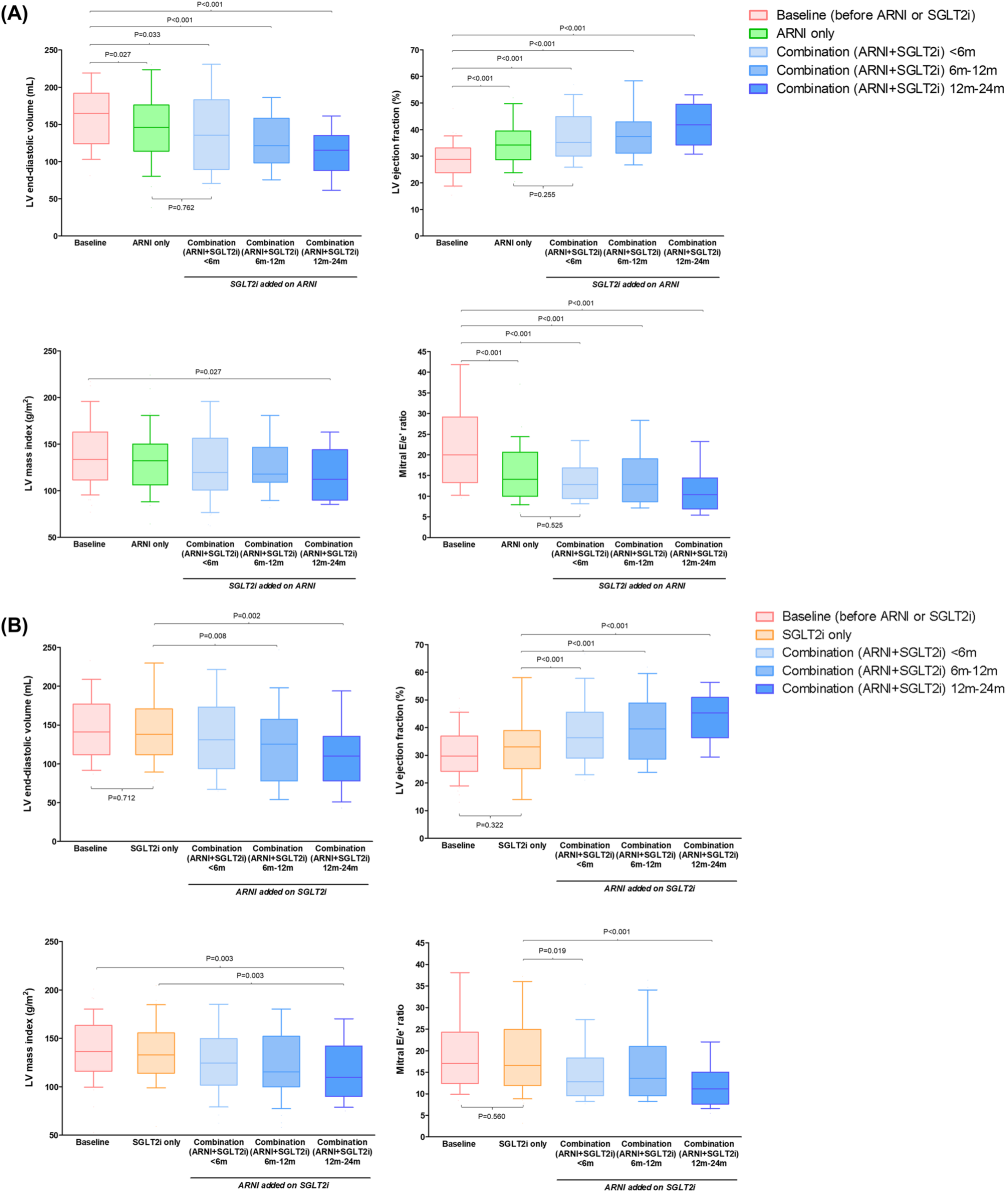
Changes in cardiac function based on the order initiation of ARNI and SGLT2i treatment. The serial measurements of LV ejection fraction, LV end-diastolic volume, LV mass index, and mitral E/e′ ratio are plotted in (**A**) patients who initiated ARNI before SGLT2i, and (**B**) patients who initiated SGLT2i before ARNI.

Since treatment with ARNI and SGLT2i was not initiated simultaneously for patients in group 1, we assessed the serial changes in echocardiographic parameters based on the order of initiation of ARNI and SGLT2i therapy. The patients in group 1 were assessed prior to propensity score matching (N = 153). The improvements in LV-EF and mitral E/e′ were found to be more pronounced in patients in whom ARNI treatment was initiated first, compared to those in whom the SGLT2i was prescribed first (Fig. 4). In the patients in whom ARNI treatment was initiated before SGLT2i, the LV-EDV decreased, the LV-EF improved, and the E/e′ ratio decreased markedly during the early period of treatment with ARNI. No significant further improvements were observed after the addition of SGLT2i. On the other hand, when SGLT2i treatment was initiated before ARNI, the improvement in cardiac function was minimal during the SGLT2i treatment period. The addition of the ARNI to the treatment regimen significantly improved the LV-EDV, LV-EF and E/e′. The more pronounced improvements in echocardiographic parameters after the addition of ARNI to SGLT2i therapy were also seen in the patients selected after propensity score matching (N = 51) (Supplementary Fig. 1).

## Discussion

In this retrospective study, we demonstrated that a combination of ARNI and SGLT2i was associated with a more significant improvement in cardiac function and a lower risk of cardiovascular death and HHF in diabetic patients with HFrEF. An increase in the LV-EF and a reduction in the mitral E/e′ ratio were observed 1–6 months after the initiation of ARNI-SGLT2i combination therapy. These improvements were larger than those seen in patients who received either ARNI or SGLT2i alone, as well as those who did not receive either of the two. It is interesting to note that the improvement in cardiac function was more prominent after the initiation of ARNI therapy, regardless of baseline SGLT2i use, compared to the vice-versa. These findings suggest that the combination of ARNI and SGLT2i could improve clinical outcomes in diabetic patients with HFrEF, and the early initiation of combination therapy may provide additional benefits.

Until the mid-2010s, RAS blockers, beta blockers, and MRA were the mainstay of the treatment of HFrEF^15^. However, the mortality and HHF remained high, creating a need for the development of new treatments^16^. The development of ARNI marked a major breakthrough in the treatment of HF, since it was able to reduce the risk of cardiovascular death by 20% and that of HHF by 21%, compared to treatment with the RAS blocker, enalapril^1^. Subsequent studies reported that the prognostic benefits by ARNI are derived from and translated to robust improvements in echocardiographic parameters^5,17,18^. Although the overall prognosis is worse in diabetic patients with HFrEF than in non-diabetic patients with HFrEF^19,20^, ARNI was observed to be beneficial even in the presence of diabetes^6^. Several mechanisms have been suggested to explain the consistent cardioprotective effects of ARNI in patients with diabetes. First, the inhibition of neprilysin increases the concentration of various vasoactive peptides including natriuretic peptide, bradykinin, angiotensin I, angiotensin II, and glucagon-like peptide. The elevated levels of vasoactive peptides improve glycemic control by increasing insulin sensitivity and metabolism, enhance the mobilization of lipids from adipose tissue, improve muscular oxidative capacity, and enhance adiponectin release. All of these are crucial for pathologic cardiac remodeling^6,21,22^. Secondly, the increased levels of cyclic guanosine monophosphate prevent the loss of protective effects of protein kinase G, which promotes diastolic relaxation, improves ventriculoatrial coupling, and blunts cardiomyocyte stiffness and hypertrophy^6,23^. The cardiovascular benefits of SGLT2i—another breakthrough class of drugs for the treatment of HFrEF—have been reported in several randomized controlled trials, especially their role in reducing the risk of composite worsening of HF^2,3,7–9^. There are several mechanisms suggested for the protective effect of SGLT2i on HF. In hemodynamic aspects, SLGT2i decreases preload and afterload, and reduces plasma and interstitial volume. In addition, SGLT2i acts on proximal renal tubule, and promotes reduction in intraglomerular pressure through restored tubule-glomerular feedback. Alleviated renal stress could improve cardiac function through reduced sympathetic nerve system activation, inflammation, and reactive oxygen species generation. Additional protective mechanisms of SGLT2i against HF are thought to be a result of improved efficiency of myocardial energy metabolism^24–27^.

Since ARNI and SGLT2i have different mechanisms of cardioprotective action, a combination of these drugs may exhibit synergistic effects. Representative trials of SGLT2i, such as DAPA-HF and EMPEROR-Reduced trials have shown consistent benefits in the treatment of HFrEF, regardless of the use of ARNI^3,10,11^. However, evidence of synergism has mostly been inferred from subgroup-analysis. In order to compare the effects of combination therapy with those of each individual drug, we divided the patients into 4 groups based on the use of ARNI and SGLT2i, and analyzed the outcomes after propensity-score matching. We found that diabetic patients with HFrEF treated with a combination of ARNI and SGLT2i showed a lower risk of HHF and cardiovascular death compared to those treated with only one or neither of these drugs. Our findings are consistent with those of prior studies, and further support the idea that ARNI and SGLT2i act through independent mechanisms and offer additional benefits in the treatment of HF.

Additionally, we compared the changes in echocardiographic parameters, which are indicative of the response to treatment, and can also translate into prognostic benefits^28^. Improvements in echocardiographic parameters were observed 1–6 months after the initiation of treatment and were maintained until 12–24 months after initiation, suggesting that the early initiation of combination therapy may result in better prognosis in diabetic patients with HFrEF. Additionally, analysis of the patients in group 1, who received ARNI-SGLT2i combination therapy, showed a more pronounced increase in the LV-EF and decrease in the mitral E/e′ ratio with the addition of ARNI to SGLT2i therapy, compared to the addition of SGLT2i to ARNI therapy. This indicates ARNI resulted in prominent LV reverse remodeling regardless of SGLT2i. These findings are consistent with those of the PROVE-HF and EVAUATE-HF trials, in which the patients treated with ARNI showed a significant improvement in the LV function parameters^5,17^. Although the additional reverse remodeling effect of SGLT2i was not significant in patients who were already receiving an ARNI, it was noted that the improvement in echocardiographic parameters and reduction in risk of cardiovascular death and HHF were more prominent in patients receiving combination therapy, than in those being treated with ARNI alone. Therefore, we believe that the inconspicuous changes in LV function after the addition of SGLT2i to ARNI therapy do not negate the cardioprotective effect of SGLT2i. Instead, this finding indicates that SGLT2i not only triggers LV reverse remodeling but also has favorable effects on HF, which may be associated with fundamental “myocardial effects”, such as myocardial energy metabolism^24,25,29^. Further studies using various imaging modalities and evaluating biomarkers, are required to investigate the effective mechanism of action of SGLT2i when it is added to ARNI treatment regimens.

This study had several limitations. Firstly, this study included a relatively small number of patients and events; thus, the results of the present study should be interpreted with caution. It was a retrospective study, without pre-scheduled echocardiography. Propensity score matching for the study population was done to shortlist the participants, and the echocardiographic measurements taken within certain time-intervals were assessed. Secondly, data on the baseline symptomatic status, such as NYHA functional class, and improvement of symptoms due to drug therapy could not be obtained due to the retrospective nature of the study. Thirdly, this study focused on the diabetic patients with HFrEF, meaning that these findings cannot be generalized to the rest of the population. Given the consistent benefits of ARNI and SGLT2i observed in non-diabetic patients with HFrEF in previous studies^2,3,6^, further studies to investigate whether our findings can be extrapolated to non-diabetic patients with HFrEF are warranted.

## Conclusions

A combination of ARNI and SGLT2i was found to reduce the risk of HHF and cardiovascular death in diabetic patients with HFrEF. More prominent improvements in LV function were observed in patients treated with the combination compared to those treated with only one or neither of these drugs. These findings suggest that the use of the ARNI-SGLT2i combination could improve the clinical course of HFrEF in diabetic patients.

## Supplementary Information

Supplementary Information.
