# Supplementary material for: Combined effects of ARNI and SGLT2 inhibitors in diabetic patients with heart failure with reduced ejection fraction
Source: Sci Rep. 2021 Nov 16;11:22342. doi: 10.1038/s41598-021-01759-5 (PMC8595580; doi:10.1038/s41598-021-01759-5)

**Supplementary material**

**Combined Effects of ARNI and SGLT2 inhibitors in Diabetic Patients with Heart Failure with Reduced Ejection Fraction**

Hyue Mee Kim^1^, In-Chang Hwang^2,3^*, Wonsuk Choi^3,4^, Yeonyee E. Yoon^2,3^, Goo-Yeong Cho^2,3^

^1^ Division of Cardiology, Department of Internal Medicine, Chung-Ang University Hospital, Seoul, South Korea

^2^ Department of Cardiology, Cardiovascular Center, Seoul National University Bundang Hospital, Seongnam, Gyeonggi, South Korea

^3^ Department of Internal Medicine, Seoul National University College of Medicine, Seoul, South Korea

^4^ Cardiovascular Center, Sheikh Khalifa Specialty Hospital, Ras Al Khaimah, United Arab Emirates

***Corresponding author:**

In-Chang Hwang, MD

Assistant Professor,

Department of Internal Medicine, Seoul National University College of Medicine,

Cardiovascular Center, Seoul National University Bundang Hospital,

82 Gumi-ro-173-gil, Bundang, Seongnam, Gyeonggi, 13620, South Korea

Tel.: +82-31-787-7065 / Fax: +82-31-787-4290

E-mail: inchang.hwang@gmail.com

**Supplementary Table 1. Baseline characteristics according to the groups**

|  | **ARNI+SGLT2i**  **(Group 1,N=166)** | **ARNI only**  **(Group 2,N=348)** | **SGLT2i only**  **(Group 3,N=89)** | **Control**  **(Group 4, N=485)** | **P value** |
| --- | --- | --- | --- | --- | --- |
| **Demographics** |  |  |  |  |  |
| Age (years) | 63.8±12.4 | 65.1±14.3 | 62.8±13.0 | 70.4±12.7 | <0.001 |
| Male (n, %) | 137 (82.5%) | 248 (71.3%) | 66 (74.2%) | 296 (61.0%) | <0.001 |
| Body mass index (kg/m^2^) | 25.1±3.6 | 24.2±3.7 | 24.8±3.4 | 23.4±4.4 | <0.001 |
| Body surface area (m^2^) | 1.8±0.2 | 1.7±0.2 | 1.7±0.2 | 1.6±0.2 | <0.001 |
| **Hemodynamics** |  |  |  |  |  |
| Systolic blood pressure (mmHg) | 118.8±16.7 | 118.8±17.7 | 122.9±22.2 | 129.3±26.9 | <0.001 |
| Diastolic blood pressure (mmHg) | 72.9±12.6 | 70.7±13.1 | 73.0±12.6 | 75.1±18.3 | 0.001 |
| **Underlying diseases (n, %)** |  |  |  |  |  |
| Hypertension | 70 (42.2%) | 105 (30.2%) | 36 (40.4%) | 323 (66.6%) | <0.001 |
| Dyslipidemia | 42 (25.3%) | 67 (19.3%) | 18 (20.2%) | 36 (7.4%) | <0.001 |
| Chronic kidney disease | 46 (27.7%) | 112 (32.2%) | 10 (11.2%) | 272 (61.8%) | <0.001 |
| Coronary artery disease | 69 (41.6%) | 126 (36.2%) | 36 (40.4%) | 232 (47.8%) | 0.009 |
| Atrial fibrillation | 53 (31.9%) | 99 (28.4%) | 33 (37.1%) | 132 (27.2%) | 0.232 |
| **Medication** |  |  |  |  |  |
| Beta blocker | 153 (92.2%) | 323 (92.8%) | 80 (89.9%) | 294 (60.6%) | <0.001 |
| MRA | 76 (45.8%) | 161 (46.3%) | 39 (43.8%) | 242 (49.9%) | 0.572 |
| Metformin | 115 (69.3%) | 37 (10.6%) | 70 (78.7%) | 99 (20.4%) | <0.001 |
| Insulin | 48 (28.9%) | 61 (17.5%) | 22 (24.7%) | 226 (46.5%) | <0.001 |
| Sulfonylurea | 49 (29.5%) | 23 (6.6%) | 31 (34.8%) | 119 (24.5%) | <0.001 |
| Antiplatelet | 100 (60.2%) | 208 (59.8%) | 53 (59.6%) | 424 (87.4%) | <0.001 |
| Anticoagulant | 67 (40.4%) | 124 (35.6%) | 33 (37.1%) | 133 (27.4%) | 0.005 |
| Statin | 132 (79.5%) | 202 (58.0%) | 65 (73.0%) | 312 (64.3%) | <0.001 |
| **Laboratory examination** |  |  |  |  |  |
| Hemoglobin (g/dL) | 13.5±2.1 | 13.4±2.0 | 13.8±2.3 | 13.0±2.3 | <0.001 |
| Hemoglobin A1c (%) | 7.4±1.4 | 6.1±0.9 | 7.5±1.6 | 6.5±1.3 | <0.001 |
| Creatinine (mg/dL) | 1.2±0.8 | 1.3±1.3 | 1.0±0.5 | 1.8±2.0 | <0.001 |
| Estimated glomerular  filtration rate (mL/min/1.73m^2^) | 74.5±24.7 | 70.3±27.2 | 78.7±28.9 | 55.8±30.3 | <0.001 |
| Total Cholesterol (mg/dL) | 141.1±36.3 | 157.2±43.4 | 153.8±42.0 | 155.5±42.6 | <0.001 |
| NT-proBNP (pg/mL) | 1428.1  (452.2-4353.5) | 1486.2  (470.4-4591.2) | 1472.2  (440.9-4007.3) | 6767.0  (2902.5-18272.4) | <0.001 |
| **Echocardiographic exam** |  |  |  |  |  |
| LV-EDD (mm) | 60.2±7.1 | 60.2±7.7 | 59.2±7.6 | 57.6±8.4 | 0.075 |
| LV-EDV (mL) | 157.9±52.5 | 164.5±58.4 | 147.5±52.1 | 133.6±51.2 | <0.001 |
| LV-EF (%) | 29.1±7.4 | 27.7±6.8 | 29.2±7.1 | 28.1±7.3 | <0.001 |
| LV mass index (g/m2) | 140.5±39.4 | 140.6±37.8 | 134.3±27.6 | 148.8±43.6 | 0.001 |

ACEI: angiotensin converting enzyme inhibitor, ARB: angiotensin receptor blocker, MRA: mineralocorticoid receptor antagonist. BNP: B-type natriuretic peptide, LV: left ventricle, EDD: end diastolic dimension, EDV: end diastolic volume, EF: ejection fraction.

Supplementary Figure 1


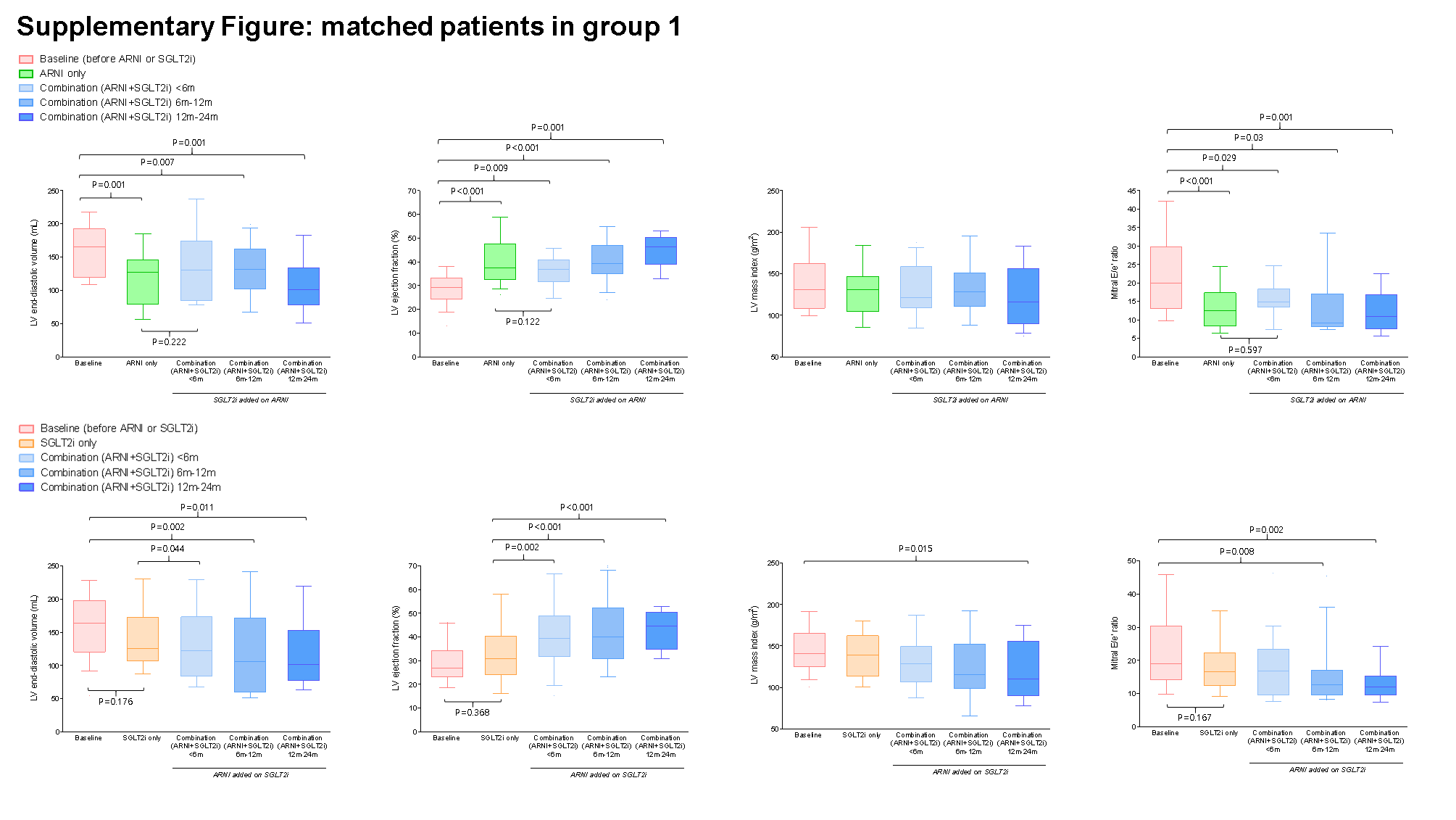

Supplement: Supplementary file 1 — Supplementary Information. [file 41598_2021_1759_MOESM1_ESM.docx]
